# Flow Control around the UAS-S45 Pitching Airfoil Using a Dynamically Morphing Leading Edge (DMLE): A Numerical Study

**DOI:** 10.3390/biomimetics8010051

**Published:** 2023-01-26

**Authors:** Musavir Bashir, Nicola Zonzini, Ruxandra Mihaela Botez, Alessandro Ceruti, Tony Wong

**Affiliations:** 1 Research Laboratory in Active Controls, Avionics and Aeroservoelasticity (LARCASE), Department of Systems Engineering, École de Technolgie Supérieure, 1100 Notre-Dame West, Montreal, QC H3C 1K3, Canada; 2 Department of Industrial Engineering, University of Bologna, Via Zamboni, 33, 40126 Bologna, Italy

**Keywords:** morphing, unsteady parameterization, Dynamically Morphing Leading Edge (DMLE), dynamic stall, flow control

## Abstract

This paper investigates the effect of the Dynamically Morphing Leading Edge (DMLE) on the flow structure and the behavior of dynamic stall vortices around a pitching UAS-S45 airfoil with the objective of controlling the dynamic stall. An unsteady parametrization framework was developed to model the time-varying motion of the leading edge. This scheme was then integrated within the Ansys-Fluent numerical solver by developing a User-Defined-Function (UDF), with the aim to dynamically deflect the airfoil boundaries, and to control the dynamic mesh used to morph and to further adapt it. The dynamic and sliding mesh techniques were used to simulate the unsteady flow around the sinusoidally pitching UAS-S45 airfoil. While the $\gamma -Re_{\theta \;}$ turbulence model adequately captured the flow structures of dynamic airfoils associated with leading-edge vortex formations for a wide range of Reynolds numbers, two broader studies are here considered. Firstly, (i) an oscillating airfoil with the DMLE is investigated; the pitching-oscillation motion of an airfoil and its parameters are defined, such as the droop nose amplitude ($A_{D}$) and the pitch angle at which the leading-edge morphing starts ($M_{ST}$). The effects of the $A_{D}$ and the $M_{ST}$ on the aerodynamic performance was studied, and three different amplitude cases are considered. Secondly, (ii) the DMLE of an airfoil motion at stall angles of attack was investigated. In this case, the airfoil was set at stall angles of attack rather than oscillating it. This study will provide the transient lift and drag at different deflection frequencies of 0.5 Hz, 1 Hz, 2 Hz, 5 Hz, and 10 Hz. The results showed that the lift coefficient for the airfoil increased by 20.15%, while a 16.58% delay in the dynamic stall angle was obtained for an oscillating airfoil with DMLE with $A_{D}$ = 0.01 and $M_{ST}$ = 14.75°, as compared to the reference airfoil. Similarly, the lift coefficients for two other cases, where $A_{D}$ = 0.05 and $A_{D}$ = 0.0075, increased by 10.67% and 11.46%, respectively, compared to the reference airfoil. Furthermore, it was shown that the downward deflection of the leading edge increased the stall angle of attack and the nose-down pitching moment. Finally, it was concluded that the new radius of curvature of the DMLE airfoil minimized the streamwise adverse pressure gradient and prevented significant flow separation by delaying the Dynamic Stall Vortex (DSV) occurrence.

## 1. Introduction

Modern Unmanned Aerial Vehicles (UAVs) have various advantages, including low operating costs, the ability to fly in risky conditions, and long flight endurance. UAVs are used for fire detection, search and rescue, wildlife monitoring, and security surveillance [1,2]. The fixed-wing UAV plays an essential role in such missions due to its long endurance and high payload capacity. However, UAVs are designed for particular missions, indicating that they can perform well for these flight conditions; however, their performance is suboptimal for their multi-point flight envelope. In addition, during various flight conditions, UAVs might experience a sudden variation of pitch attitude due to the necessity of performing extraordinary maneuvers or to unexpected external disturbances, such as vertical wind gusts. Consequently, an abrupt and sudden change in the angle of attack may develop non-linear unsteady aerodynamic effects such as dynamic stall on the aircraft.

Furthermore, UAVs are currently holding most of the market share [3], and expect to contribute up to 20% of the global aviation market by 2037. Therefore, designers are encouraged to develop breakthrough technology to meet the Green Aviation standards, and reducing fuel consumption has become very important for the environment and air transportation. The challenges of meeting the demands for lower emissions and higher levels of air transport have increased the demand for new research ideas to produce more efficient and ecologically friendly aircraft.

Researchers have attempted to mimic bird flight to improve the aerodynamic performance of a broader flight envelope. However, such an attempt requires a high level of technology readiness, which allows it to be installed on UAVs because of safety and weight concerns [4,5]. This possibility of meeting the desired aerodynamic efficiency by mimicking the bird flight is the new generation morphing wing technology. The early years of aeronautical research focused on biomimetic techniques based on flexible and highly deformable structures to mimic the ability of birds to adjust their shape to different flight cases. The Research Laboratory in Active Controls, Avionics, and AeroServoElasticity (LARCASE) team is studying several approaches for reducing fuel consumption [6,7,8,9,10,11,12,13,14,15,16], including the use of morphing wing technologies, such as the morphing leading edge prototype demonstrates the possibility of modifying the stall angle of the wing. It was shown that the stall angle of the wing was delayed using a downward deformation of the leading edge [17,18]. An experimental and numerical analysis was carried out for a UAS-S45 wing geometry with the aim to improve the laminar flow on the upper surface of the wing, between 10% and 70% of the chord [19,20,21]. The morphing optimization considered different flight conditions, such as take-off, cruise, landing, stall, etc. It was shown that for all investigated cases, a significant transition improvement was obtained.

Traditional hinged lifting mechanisms and trailing edge surfaces control airflow aerodynamically, but they also increase drag [22]. These hinged surfaces have drawbacks in both deployed and retracted states [23]. When hinged surfaces are deployed, the gaps between the high-lifting surface and the wing can create noise, turbulence, and early transition. Retracted trailing edge hinges provide a turbulent boundary layer. Various techniques have been implemented recently to enhance aerodynamic efficiency. One such solution is the morphing wing technology, a promising state-of-the-art innovation [24,25,26]. Several research programs have achieved significant results in the field of aircraft morphing [27,28,29].

The use of morphing wing technology as a flow control technology has resulted in efficient aerodynamic designs [30,31,32,33]. Dynamic stall control is especially significant because it occurs in all aerospace applications, such as on UAVs [34,35], helicopter rotors [36,37], wind turbines [38,39], military aircraft [40,41], and others. Researchers have focused on tackling the dynamic stall phenomenon experienced by pitching oscillating airfoils for several years [42,43,44,45,46]. Dynamic stall is caused by a rapidly pitched airfoil, wing, or turbine blade. The Leading-Edge Vortex (LEV), or Dynamic Stall Vortex (DSV), is a critical component of dynamic stall, as it increases the lift coefficient. However, this excess lift is lost when the vortex sheds into the wake, thus increasing drag and shifting the pitching moment. Since the discovery of dynamic stall, researchers have tried to understand its mechanics and to modify the LEV formation. Altering the LEV formation may increase an aircraft’s operational envelope.

Numerous flow control devices have been developed to avoid flow separation and to mitigate dynamic stall effects. They can be classed into active and passive, based on their operational principles. Vortex generators, micro-tabs, and serrated trailing edges are all examples of passive control mechanisms, while active control mechanisms include trailing-edge flaps. synthetic jets, plasma actuators, etc. The use of leading-edge slats [47,48], trailing-edge flaps [49,50], synthetic jet/periodic excitations [51,52,53], plasma actuators [54], vortex generators [55], and dynamically deformed leading edges [56] to accomplish the flow control has been studied.

Several studies have been carried out to investigate the aerodynamics of morphing wings and their stall properties. The Reynolds-Averaged Navier-Stokes (RANS) equations were used to analyze adaptive morphing trailing-edge wings, thus producing a drag reduction on-design of 1%, and 5% off-design [57]. The lift-drag ratio increased by 6.5% for the morphing wing [58]. The numerical simulations of a NACA0012 airfoil with a flexible trailing edge showed that the morphing surface could postpone the beginning of flow separation with the aim to achieve optimal aerodynamic performance [59,60].

An active dynamic stall control technique with deployable LEV generators was developed in [61]. For a dynamic stall flight, the active flow control would only be triggered on the retreating side of the blade to avoid increasing blade drag. The vortex generators performed very well in the wind tunnel, as they delayed the static stall and reduced the dynamic stall penalties. The static stall angle increased by three degrees, and the negative pitching moment peak was lowered by 60% for the dynamic stall.

The impact of synthetic jet control on the unstable dynamic stall over a rotor airfoil using numerical simulations was explored in [62]. The numerical findings indicated that the dual jet could significantly increase the control efficiency of the rotor airfoil’s dynamic stall compared to the single jet. A high-frequency control method for dynamic stall mitigation that takes advantage of the natural instabilities of the laminar separation bubble (LSB) to delay the occurrence of Dynamic Stall Vortex (DSV) was proposed in [63,64] and merits further investigation. The effect of the trailing edge flap (TEF) on mitigating DSV-induced substantial negative pitching moments and negative aerodynamic damping was investigated in [65]. The authors hypothesized that the substantial negative pitching moments and related negative aerodynamic damping were caused by the TEV. The effect of the oscillating TEF on the dynamic loads generated by an airfoil was evaluated [66,67]. The TEF motion did not affect the creation or separation of the DSV, but the TEF’s deflection increased the airfoil’s maximum lift.

A study investigating the effect of a TEF on the dynamic stall at high speeds for wind turbines was presented in [68]. The TEF pitching impacts the load fluctuation loops. The trailing edge flap reduced cyclic variability by 26% in terms of root bending moment. These findings help in better understanding how TEFs reduce wind turbine blade load changes. Unsteady aerodynamic loads were applied on an airfoil by a TEF deflecting at various frequencies [69]. Phase delay between an airfoil angle of attack and the initial flap deflection was investigated. When the airfoil’s angle of attack and TEF deflection both increased, the TEF oscillations increased the maximum lift coefficient.

A hybrid RANS-LES technique was used to investigate the airfoil morphing aerodynamic performance with a deflecting TEF [70,71]. Morphing enhanced the lift-to-drag ratio by 6%. Similar type results were obtained for the aerodynamic and aero-acoustic responses of an airfoil fitted with a harmonically morphing TEF [72]. Larger morphing TEF amplitudes increased Sound Pressure Levels (SPLs), and all morphing cases studied shifted the main tonal peak to a higher frequency that led to a 1.5 dB reduction in the predicted SPL.

A two-dimensional multi-element dynamic stall solver was investigated to demonstrate the leading edge slat’s effectiveness in controlling dynamic stall [73]. The major drawback of these slats was their high drag penalties associated with their use under off-design conditions. A retraction mechanism similar to that found on aircraft would be practically heavy and expensive.

Since the local shape of the airfoil leading-edge influences DSV production, changing the leading-edge shape is an efficient technique to reduce dynamic stall effects [74]. Active flow control based on a Variable Droop Leading-Edge (VDLE) device reduced local Mach number and improved pressure distribution near the leading edge, therefore postponing or eliminating dynamic stall without significant lift changes [75]. The concept of a dynamically deforming leading edge was proposed, where the airfoil shape was gradually changed, and the leading-edge radius increased as the airfoil pitched upwards [76]. Airfoils with large leading-edge radii tend to have mild adverse pressure gradients, as the local velocities are lower than those of a conventional airfoil. As the airfoil pitches downwards in the absence of a stall the airfoil returns to its original shape. In another study, the leading-edge droop was integrated on the Gurney flap to improve rotor airfoil dynamic stall and post stall [77]. The deflection of a 20° leading-edge droop with that of a 0.5% chord Gurney flap effectively delayed dynamic stall. The maximum lift coefficient increased, while the negative pitching moment decreased, and the lift-to-drag ratio increased with respect to reference designs.

The potential benefits of controlling and further delaying this nonlinear aerodynamic stall effect could be achieved by employing a Dynamically Morphing Leading Edge (DMLE). An in-depth understanding of the unsteady flow physics characterizing this class of morphing wings would be helpful. The DMLE concept as a comprehensive investigation of different parameters, such as the deflection frequency, the extent of deflection, and the morphing starting time, is barely found in the literature. This study aims to track the occurrence and development of an LEV and reduce the flow separation, thereby, avoiding the dynamic stall. The dynamic stall control with the DMLE on an oscillating UAS-S45 airfoil is evaluated using unsteady RANS equations. Two different studies and analyses are proposed and investigated.

## 2. Methodology

This study investigates the effect of a Dynamically Morphing Leading Edge (DMLE) on the dynamic stall of the UAS-S45 airfoil. The influence of the upward and downward airfoil deflections on the development process of the Dynamic Stall Vortex (DSV) is presented for the oscillating airfoil. To simulate the dynamic stall phenomenon, the pitching of the airfoil is governed by a time-dependent sinusoidal equation that guarantees a bounded time variation of the angle of attack. The pitching motion results in the generation of a periodic hysteresis cycle of the aerodynamic coefficients, including the lift, drag, and moment coefficients. The following sinusoidal mode Equation (1) governs the airfoil pitching motion about its ¼ chord position:

(1) $$\alpha \left(t\right)=\alpha_{m}+\alpha_{a}\sin\left(\omega t\right)$$

where $\alpha_{m}$ = 11° is the mean incidence angle, $\alpha_{a}$ = 20° is the amplitude, ω is the angular velocity and *t* is the time.

The reduced frequency (k) is defined in Equation (2):

(2) $$k=\frac{\omega c}{2U_{\infty }}$$

where $U_{\infty }$ is the freestream velocity and *c* is the airfoil chord length. A reduced frequency of *k* = 0.08 was chosen for the airfoil pitching motion and the Reynolds number based on the unit chord length and free-stream velocity of 35.5 m/s was set to 2.4 × 10^6^.

### 2.1. Leading Edge Parametrization

**Figure 1:**
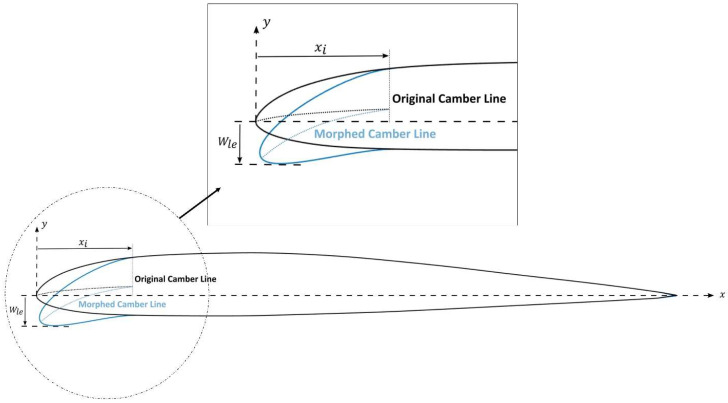
Geometrical definition of a variable camber line.

The morphing leading edge geometry and its unsteady deformation must be defined in order to obtain the DMLE airfoil. These definitions require a parametrization technique that can accurately characterize the airfoil boundary/geometry. By using specific control parameters from the 4-digit NACA airfoil, it was possible to dynamically adjust the camber line of the targeted morphing region of the chord and to design a new airfoil shape that included the time variable in the parametrized equations of the trailing edge geometry [78]. However, due to the UAS-S45 airfoil asymmetry, it was impossible to directly adopt and converse the mathematical model of symmetric airfoil [78]. Therefore, a different concept was developed here for the asymmetric airfoils. The camber of an asymmetric airfoil determines its curvature and its type. Figure 1 shows the parameters considered in the leading-edge morphing model. The beginning of the morphing region is given by the parameter $x_{i}$, and the maximum displacement of the outermost leading-edge coordinate is given by $W_{le}$.

The essential requirement for designing a DMLE framework is the development of the parametric equations for the camber line and the airfoil thickness distribution. These equations are developed for the asymmetric UAS-S45 airfoil model and are therefore used in the present study. The airfoil thickness distribution is given by Equation (3):

(3) $$\frac{y_{t}}{c}=\left(\frac{t}{c}\right)\;\left[a_{0}\sqrt{\frac{x}{c}}-a_{1}\left(\frac{x}{c}\right)-a_{2}{\left(\frac{x}{c}\right)}^{2}+\;a_{3}{\left(\frac{x}{c}\right)}^{3}-a_{4}{\left(\frac{x}{c}\right)}^{4}\right]$$

Depending on the airfoil part being considered (Leading-edge or Trailing edge) and on the chosen starting point of its morphing, it is possible to use Equation (4) or Equation (5):

(4) $$\begin{array}{c} \frac{y_{c}}{c}=\frac{M}{P^{2}}\left[2P\left(\frac{x}{c}\right)-{\left(\frac{x}{c}\right)}^{2}\right]\;\;\;\;\;\\ \frac{dy_{c}}{dx}=\frac{2M}{P^{2}}\left[P-\left(\frac{x}{c}\right)\right] \end{array}\}\;\;\;\left(\frac{x}{c}\right)<P\;$$

(5) $$\begin{array}{c} \frac{y_{c}}{c}=\frac{M}{{\left(1-P\right)}^{2}}\left[1-2P+2P\left(\frac{x}{c}\right)-{\left(\frac{x}{c}\right)}^{2}\right]\\ \frac{dy_{c}}{dx}=\frac{2M}{{\left(1-P\right)}^{2}}\left(P-\left(\frac{x}{c}\right)\;\right) \end{array}\;\;\;\}\;\left(\frac{x}{c}\right)\;\geq P\;\;$$

where *M* is the maximum value of the percentage chord line, and *P* is the chordwise position of the maximum camber in 10′s of the chord. For the definition of the camber line, since *M* and *P* for an asymmetric airfoil are not zero, the value of *P* serves to divide the camber line approximation into two separate equations in which both *M* and *P* appear.

After the definitions of the airfoil mean camber line in Equation (4) or Equation (5), a second-order polynomial function is introduced to define the new camber line equation of the morphing airfoil portion. In order to obtain control of the maximum deflection of the leading edge, Equation (6) is parametrized and defined as follows:

(6) $$\frac{y_{f}}{c}=\left\{\begin{array}{c} \frac{y_{f}}{c}-W_{le}\frac{{\left(x_{i}-\bar{x}\right)}^{2}}{x_{i}^{2}},0\leq \bar{x}\leq x_{i}\\ \;\;\;\;\;\;\;\;\;0,\;\;\;\;\;\;\;\;\;\;\;\;\;\;\;\;\;\;\;\;x_{i}\geq \;\bar{x} \end{array}\right\}$$

where $y_{f}$ is the final y-coordinate of the new morphing airfoil camber line, $W_{le}$ is the value of the maximum deflection of the leading edge, and $\bar{x}$ is the x-coordinate of the selected control point. The maximum limit of leading-edge deflection was set to 0.05c and the airfoil was initially ($x_{i}$) set at baseline position.

To achieve a continuous deflection of the leading edge during transient flow simulations, the time variable must be introduced in the governing equations of the parameterization model. The parametrization equation was extended to include the time variable, while the parametrized camber line equation was modified with the aim to express the camber line as a function of time. The unsteady dynamic motion of the upper and lower surface airfoil coordinates is obtained by adding the thickness distribution to the time-dependent camber line equation. Moreover, the airfoil deflecting motion was designed to start from the baseline configuration, then reach a maximum downward target position. It will then return to its initial baseline configuration, and a sinusoidal function has been chosen and further used. The parametrized time-dependent camber line is defined by the following equation:

(7) $$\frac{y_{f}}{c}=\left\{\begin{array}{c} \frac{y_{f}}{c}-W_{LE}\sin\left(2\pi tf\right)\frac{{\left(x_{i}-\bar{x}\right)}^{2}}{x_{i}^{2}},0\leq \bar{x}\leq x_{i}\\ \;\;\;\;\;\;\;\;\;0,\;\;\;\;\;\;\;\;\;\;\;\;\;\;\;\;\;\;\;\;x_{i}\geq \;\bar{x} \end{array}\right\}$$

where *t* is the time variable, and *f* is the airfoil deflection frequency (cycles per second).

**Figure 2:**
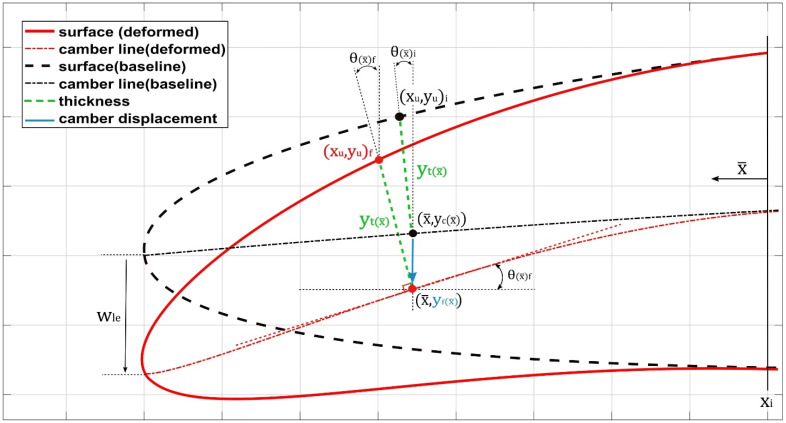
Numerical modeling of a camber line.

Five main parameters were determined to be of crucial importance for the airfoil morphing control: *M*, *P*, $y_{f}$, $W_{le}$ and $x_{i}$. Figure 2 depicts some of these parameters.

### 2.2. Computational Domain and Grid Definitions

**Figure 3:**
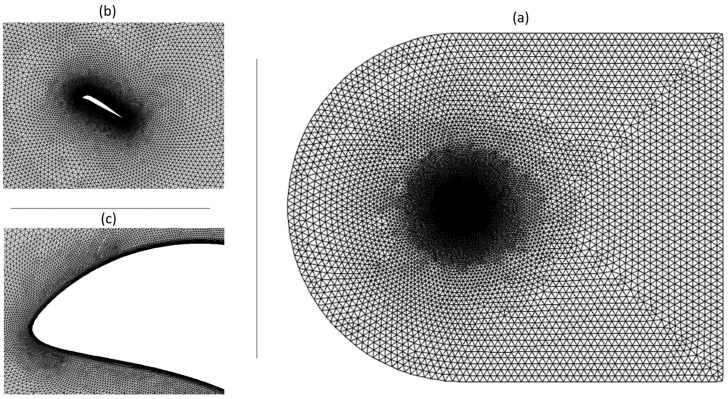
Computational domain with (**a**) hybrid mesh, (**b**) structured mesh around the airfoil, and (**c**) DMLE UAS-S45 airfoil mesh.

The size and shape of the computational domain affect the quality of results, which is also based on the geometry’s aerodynamics. For the present aerodynamics scenario, the 2D computational domain is represented by a C-shaped grid with 20 times the chord length (20c) upstream and 30 times the chord length (30c) downstream. Such lengths were found to be large enough to ensure that the outer domain boundary conditions do not influence the airfoil flow and that they are small enough to keep the computing power in a reasonable range. In this study, both unstructured and structured hybrid meshes were considered, as illustrated in Figure 3, with a structured quadrilateral layer mesh near the airfoil and with an unstructured triangle mesh elsewhere. With a blunt trailing edge, it is possible to obtain O-shaped block layers surrounding the airfoil and thereby minimize any instabilities caused by sharp trailing-edge corners.

Wall condition parameter $y^{+}$ defines a dimensionless height of the first grid point measured from a wall, that is utilized to evaluate the ‘near-wall’ mesh requirements. To appropriately represent the near-wall mesh, a grid that meets a turbulence model’s wall $y^{+}$ requirements are obtained. In the current investigation, the $\gamma -Re_{\theta }$ turbulence model required that the first airfoil cells should be situated in the viscous sublayer; thus, a $y^{+}$ less than one was targeted.

### 2.3. Validation of Results

**Figure 4:**
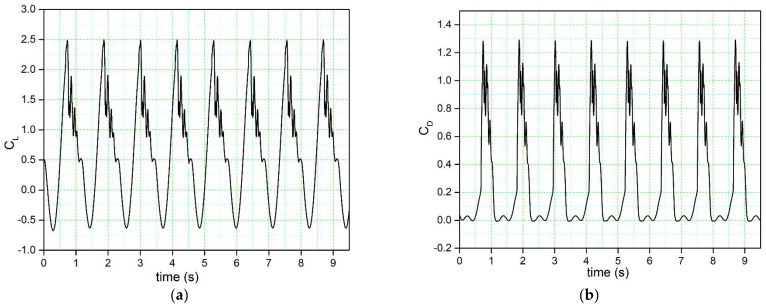
Time history of the (**a**) lift coefficient and (**b**) the drag coefficient.

The time history variation of the lift coefficient illustrated in Figure 4a displays a sudden stall after the maximum value of the lift coefficient (peak). Immediately afterward, a downstroke phase is characterized by the unsteady behavior of the flow. This trend qualitatively agrees with dynamic stall lift coefficient behavior. To better assess the simulation convergence, the lift coefficient and drag coefficients for nine pitching cycles are shown in Figure 4 and it shows that a very negligible difference is observed and the standard deviation between two successive pitching cycles was evaluated.

The outcome of this evaluation highlighted a little difference between the lift and drag coefficients between the successive pitching cycles. The difference between the force coefficients of the second cycle versus those of the rest of the cycles was negligible, approximately 0.01%. In addition, a maximum $y^{+}$ factor of 0.96 was found along the airfoil surface on the second pitching cycle, thus securing a correct first layer’s height.

**Figure 5:**
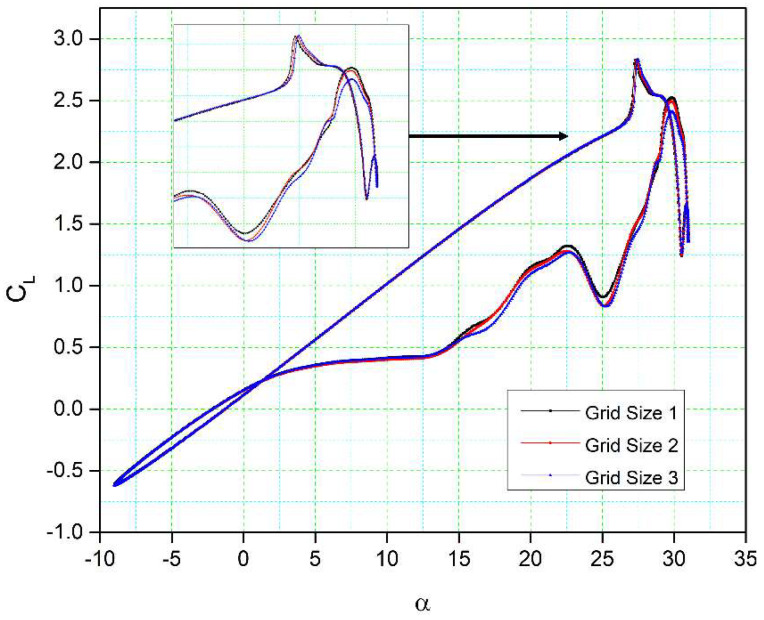
Comparisons of the numerical results for the lift coefficient versus the angle of attack for three different grid sizes.

Table 1 presents the properties of three different grid sizes used for grid independency investigations. Figure 5 shows the lift coefficient variations with the angle of attack for these three grid sizes. There is a good correlation between these results. The lift coefficients obtained for grid size 1 and grid size 2 are slightly different in the downstroke phase, while their stall values (the peak of the lift curve) have a slight variation from each other. Additionally, the fluctuations in the lift coefficient for all three grid sizes are similar in shape and magnitude. Only very small lift coefficient discrepancies are visible between grid size 3 and the other two grid sizes during the upstroke, while a small difference can be seen between the lift coefficients in the downstroke. The flow reattachment location for all three grid sizes is the same. Therefore, grid size 2 was selected as the computational domain due to its acceptable (medium-sized) cell number and overall good results.

**Figure 6:**
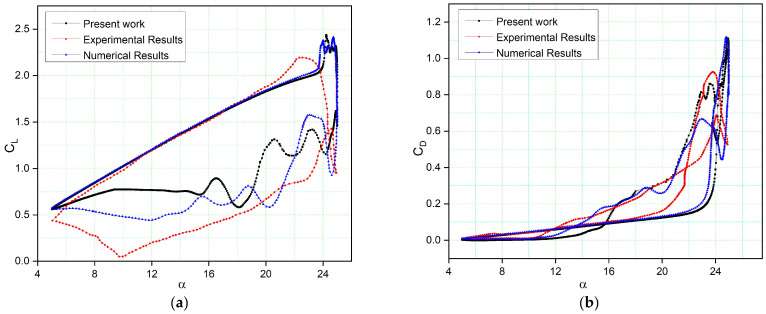
Comparison of our numerical results with experimental results obtained from wind tunnel tests [79] and numerical results [80]: (**a**) lift coefficient; (**b**) drag coefficient variations with the angle of attack.

Figure 6 compares the NACA 0012 airfoil model’s computed lift and drag coefficients to their experimental values [79] and numerical results from the previous literature [80] for the Reynolds number 2.5 × 10^6^, the reduced frequency *k* = 0.10, and angles of attack of 5° to 25°, with a mean incidence of 15° were validated while the second simulation used the numerical data from [80] for the same settings.

The $\gamma -Re_{\theta \;}$ turbulence model can forecast the results’ trend. The lift coefficient corresponds to its experimental value in the upstroke phase, but it predicts the stall differently than its experimental value. As the unsteady analysis is often dissipative, it reduces the flow intensity and thus the kinetic energy. The numerical result is good, as it captures the load variation trend before the stall region. The range of differences in the peaks of the lift coefficient is small. The downstroke variations are due to the extensive post-stall process, and they result in a discrepancy in the initial LEV prediction.

Figure 6b illustrates the drag coefficient variation with the angle of attack, revealing the notable difference when α > 12, as the deep stall causes the drag to increase. In numerical simulations [80], the drag coefficient with the angle of attack is lower than the drag coefficient obtained in other simulations. However, our numerical results of an airfoil’s maximum drag coefficient are lower than the experimental results due to large vortices on the airfoil surface and to the flow’s three-dimensionality. These vortices occur because of the persistent flow separations at high angles of attack, which makes it difficult to effectively describe the viscous effects near the airfoil surface. The CFD simulations in our study also indicate a secondary LEV that contributes to the recovery of the lift and drag coefficients around the maximum angle of attack.

## 3. Discussion of Results

This study examines the unsteady aerodynamic characteristics obtained by the same oscillating baseline airfoil equipped with a variable morphing leading edge. Since an oscillating function controls the temporal deformation of the leading edge, a frequency parameter must be considered. The pitching-oscillation motion is invariably controlled by Equation (1) where α_mean_ = 11°, α_amp_ = 20° and a reduced frequency of *k* = 0.08. The operating Reynolds number used was 2.4 × 10^6^ with a freestream velocity of U_∞_ = 35.5 m/s and a turbulence intensity of Tu = 0.1%.

Two broader studies are considered (i) The Dynamically Morphing Leading-Edge (DMLE) of an oscillating airfoil is investigated first. In this study, the pitching-oscillation motion of an airfoil is defined, and the parameters such as the droop nose amplitude ($A_{D})$, and the morphing starting time ($M_{ST}$) in terms of pitching angles are carefully chosen. The effects of the variation of $A_{D}$ and of $M_{ST}$ on the aerodynamic performance of the airfoil are studied, and three different amplitude cases considered are $A_{D}$ = 0.01, 0.005, and 0.0075. Furthermore, the combinations of $A_{D}$ and $M_{ST}$ are evaluated with the aim to find optimal results; (ii) The Dynamically Morphing Leading-Edge (DMLE) of an airfoil settled at stall angles of attack is investigated. In this case, the airfoil does not oscillate, but only the DMLE motion is studied at stall angles of attack. This analysis is performed to provide further insights into the transient lift and drag forces for the deflection frequencies of 1 Hz, 2 Hz, 5 Hz, and 10 Hz.

### 3.1. Results for an Oscillating Airfoil with Dynamically Morphing Leading Edge (DMLE)

**Figure 7:**
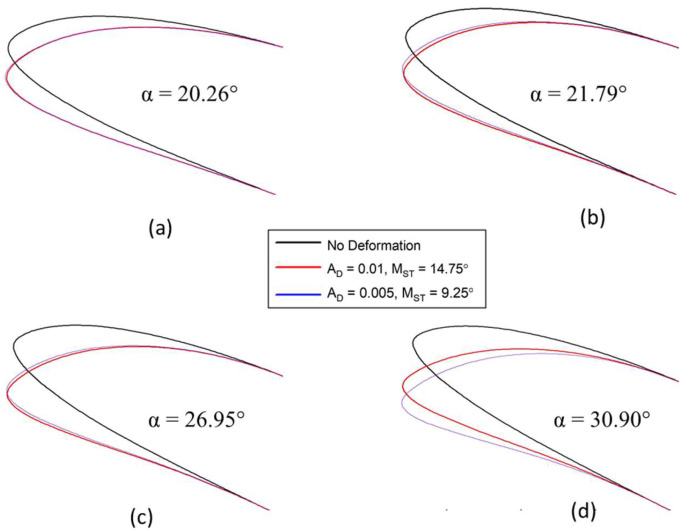
The reference airfoil in comparison with the DMLE airfoils; (**a**) both the dynamically morphing airfoils are overlapping each other at pitch angle of 20.26°, (**b**) the airfoil with $A_{D}$ = 0.01 deflects more even the morphing starts after $A_{D}$ = 0.005 at pitch angle of 21.79°, (**c**) the deflection of $A_{D}$ = 0.005 airfoil is increasing and (**d**) the deflection of $A_{D}$ = 0.005 airfoil is large as compared to $A_{D}$ = 0.01 because it continues to morph till higher pitch angles. These figures show that the droop nose amplitude and morphing starting time have significant effect on the airfoil leading edge shape.

Numbered The parameter $A_{D}$ determines the deformation extent along the airfoil chord. The different airfoil deformations for different values of $A_{D}$ are shown in Figure 7. These deformations indicate that the values of $A_{D}$ vary with the angle of attack.

**Figure 8:**
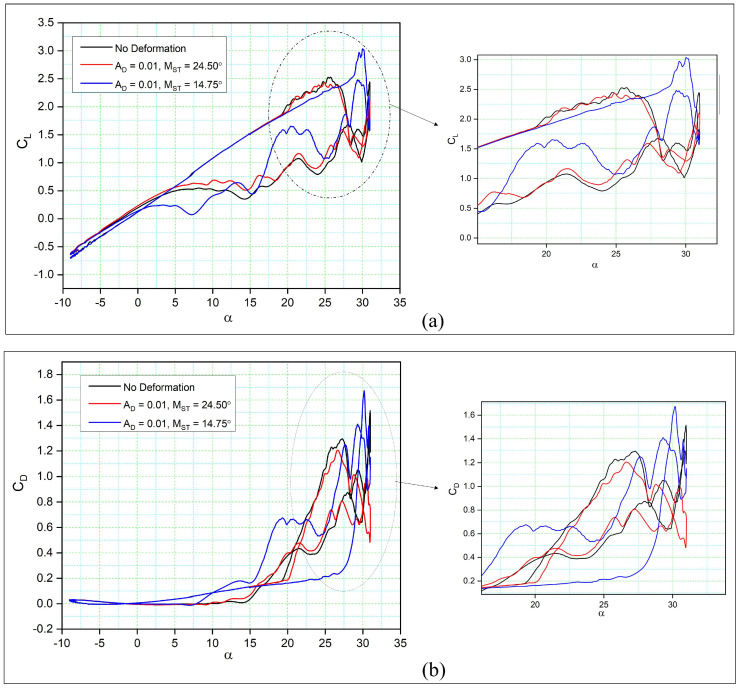
Hysteresis cycles for (**a**) the lift coefficient of DMLE airfoils and (**b**) the drag coefficient of DMLE airfoils in comparison to the reference (no deformation) airfoil.

Figure 8 compares the aerodynamic coefficients of the dynamically morphing leading-edge airfoils to those of a reference airfoil with respect to the angle of attack over one complete hysteresis cycle. Figure 8a shows that only the case with $M_{ST}$ = 14.75° has successfully increased $C_{L,max}$ with respect to the reference airfoil. It is also clear that in this case, while the morphing leading edge has increased $C_{L,max}$, it also has a delayed stall angle of attack. The main objective of this study is to delay or increase the stall angle since its delay helps to maintain favorable lift coefficient values even in the downstroke phase of the cycle. However, this result indicates that a strong vortex has been formed in the leading edge of the airfoil, which later results in the dynamic stall. Instead, for $M_{ST}$ = 24.50°, both upstroke and downstroke motions exhibit a similar trend to the baseline oscillating airfoil. This behavior clearly shows the importance of choosing an adequate $M_{ST}$ for the airfoil morphing deformation. In fact, by assuming an $M_{ST}$ very close to the end of the upstroke would not allow the leading edge to deform sufficiently, thereby no major flow variation over an airfoil is observed.

For the $M_{ST}$ = 14.75°, the lift coefficient increases almost linearly until 28°, after which an increase in the $C_{L}$ is observed. As shown in the lift coefficient curves in Figure 8a, the flow reattachment occurs early in the case of the reference (no deformation) airfoil, and for the airfoil with $M_{ST}$ = 24.50°. In fact, at the angle of 28°, these two airfoils exhibit very low lift coefficient values due to the already separated leading edge vortex. Beneficial behavior, in terms of $C_{L,max}$ increase and a delay of $\alpha_{stall}$ delay is obtained if the deformation starts early by allowing the LEV to keep increasing on the suction side of the airfoil.

Similarly, the drag coefficients are lower for both DMLE airfoils at an angle of attack (AoA) smaller than 30°, and the $C_{D,max}$ increases substantially at the peak angle with respect to the reference airfoil only at $M_{ST}$ = 14.75°, as seen in Figure 8b. Therefore, the drag coefficient of the DMLE airfoil remains low for a large range of angles of attack. After its comparison with the reference airfoil that has no deformation, the airfoil at $M_{ST}$ = 24.50° show similar behaviour. However, at $M_{ST}$ = 14.75°, the DMLE is not very efficient in reducing the drag values at stall angles of attack.

**Figure 9:**
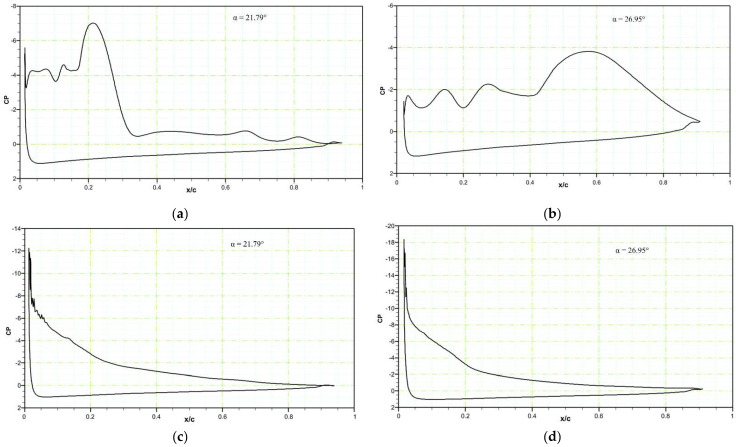
Pressure coefficients of the (**a**) reference airfoil at an AoA = 21.79°, (**b**) reference airfoil at an AoA = 26.95°, (**c**) the DMLE airfoil at an AoA = 21.79°, and (**d**) the DMLE airfoil at an AoA = 26.95°.

When AoA = 21.79°, the reference airfoil develops a strong LEV, seen as a “bump” in the surface pressure distribution in Figure 9a. When the angle of attack reaches an AoA = 26.95°, the LEV has increased in size and spread over a large part of the airfoil upper surface. There is a rapid reduction in the leading-edge suction peak due to the separation of the vortex. This shedding is accompanied by a large, abrupt decrease in the lift coefficient, as indicated in Figure 9a.

For an airfoil with $A_{D}$ = 0.01 and $M_{ST}$ = 14.75°, no strong LEV is visible. In Figure 8a, at an upstroke with an AoA = 20.3°, the flow evolves slowly and remains attached to the airfoil with a small LSB starting to form. When AoA = 25.5°, the LEV develops along the leading edge and continues to grow slowly. The increase of the angle of attack to an AoA = 27.3° increases the LEV size.

**Figure 10:**
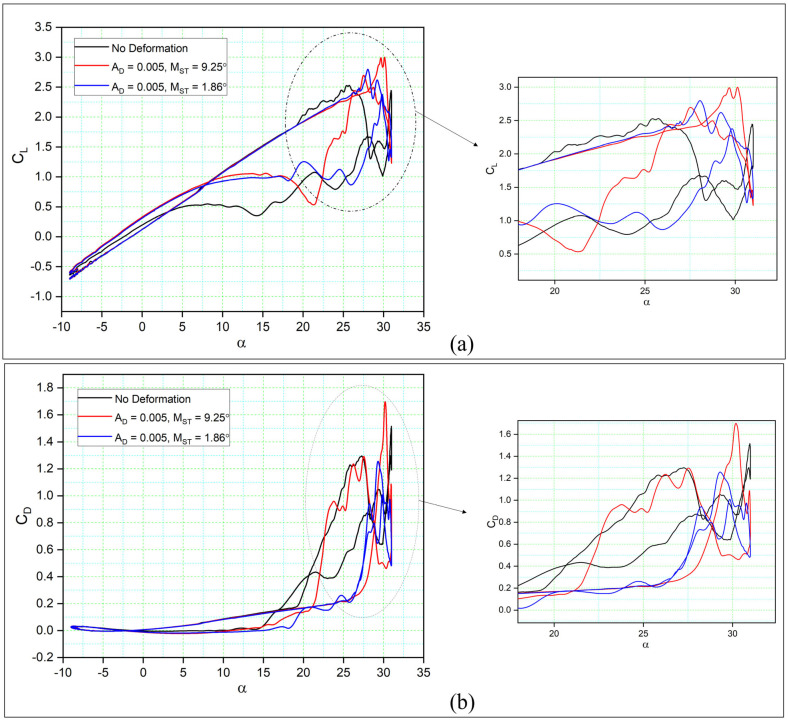
Hysteresis cycles for (**a**) the lift coefficients of DMLE airfoils and (**b**) the drag coefficients of DMLE airfoils, presented in comparison to the reference (unmorphed) airfoil.

Figure 10 compares the aerodynamic coefficients of two different $M_{ST}$ cases of DMLE airfoils to those of a reference airfoil with respect to the angle of attack over one complete cycle. Low value of $A_{D}$ ($A_{D}$ = 0.005) and $M_{ST}$ were chosen for these cases. Figure 10a shows that the DMLE airfoils both increased their $C_{L,max}$ values by 22.48% from 2.49 to 3.04 (case $M_{ST}$ = 9.25°), and from 2.49 to 2.62 (case $M_{ST}$ = 1.86°) with 5.22%. It is also clear that the DMLE increased the $C_{L,max}$ with the increase in the stall angle of attack; that was one of the significant goals of this study, because of the fact that the stall angle of attack delay helps to maintain the airfoil lift coefficient value, even in the downstroke cycle. However, this result indicates that there is a strong flow separation once the downstroke starts.

As seen in Figure 10a, where $A_{D}$ = 0.005, compared to the previous cases where $A_{D}$ = 0.01, the lift coefficient of the new DMLE airfoils increases slightly for high angles of attack. In both these cases, the lift coefficients are higher as the angle of attack increases, and therefore stall is delayed. As seen from the lift coefficients variation curves, the flow reattachment occurs earlier for the reference airfoil and the DNLE airfoil with $M_{ST}$ = 1.86°, which is the opposite behavior than that observed in the previous analysis for $A_{D}$ = 0.01. In this case ($M_{ST}$ = 1.86°), initiating the deformation early in the upstroke phase influences the flow field and thus results in a premature LEV separation.

Similarly, the drag coefficients are low for the DMLE with the $M_{ST}$ = 1.86° for angles of attack smaller than 30° than the drag coefficients of the reference airfoil. However, the $C_{D,max}$ for the other case ($M_{ST}$ = 9.25°) increases substantially for the same angles of attack with respect to the $C_{D,max}$ of the reference airfoil, as seen in Figure 10b. The sudden $C_{D,max}$ increase of this latter case can be attributed to the corresponding increase in lift, developed as a consequence of the ongoing LEV. On the other hand, when compared with the reference airfoil, the $M_{ST}$ = 1.86° case shows a smaller $C_{D}$ trend. In this case, the DMLE is able to drastically reduce the drag over the entire pitching cycle.

**Figure 11:**
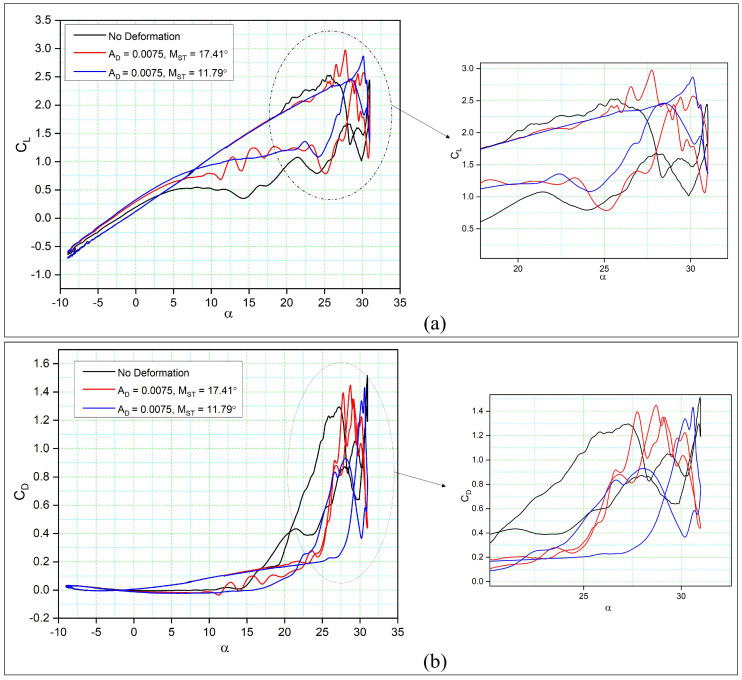
Hysteresis cycles for (**a**) the lift coefficients of DMLE airfoils and (**b**) the drag coefficients of DMLE airfoils in comparison to those of the reference (unmorphed) airfoil.

Similarly, it can be seen from Figure 11a that the maximum lift coefficient ($C_{L,max})$ of the DMLE airfoils increases slightly for high angles of attack. In addition, the flow reattachment for DMLE airfoils occurs earlier as the transition point moves from the trailing towards the leading edge. It thus appears that the DMLE airfoil performance increases during the downstroke. At an angle of attack of 10 degrees, the flow field attaches to both airfoils. The values of drag coefficients of DMLE airfoils at high angles of attack are lower than those of the reference airfoil, as shown in Figure 11b.

**Figure 12:**
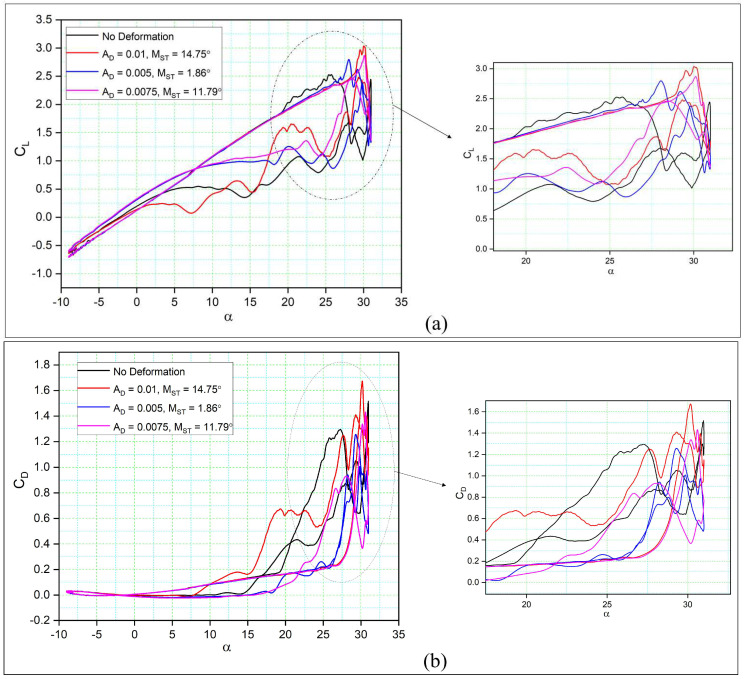
Hysteresis cycles for (**a**) the lift coefficients of DMLE airfoils and (**b**) the drag coefficients of DMLE airfoils compared to the reference airfoil.

Figure 12a compares the aerodynamic coefficients for three different amplitudes ($A_{D}$) to those of a reference airfoil. It is clear that the different amplitudes result in higher $C_{L}$ values for all the DMLE airfoils during the upstroke. At lower values of $A_{D}$ ($A_{D}$ = 0.01) and $M_{ST}$ = 14.75°, the $C_{L,max}$ reaches 3.04, while the reference airfoil’s $C_{L,max}$ is 2.53, showing a 20.15% increase in $C_{L,max}$. However, the flow reattachment for this case of the DMLE airfoil is delayed compared to that of the reference airfoil during the downstroke. During the pitching oscillation of the reference airfoil, the flow separation and stall occur at an angle of attack of 25°. However, with the droop deformation of the leading edge, the stall occurs at an angle of attack of 30°, thereby delaying the stall by 5°.

In the case when $A_{D}$ = 0.005, slightly higher lift coefficients are found, as $C_{L,max}$ increased from 2.53 to 2.8, with a 10.67% increase in comparison to the reference airfoil (Figure 12a). As the value of $A_{D}$ varies with the morphing time ($M_{ST}$), the main part of the droop deformation is closer to the leading-edge point. In case of $A_{D}$ = 0.05, the stall angle is delayed 12% in comparison to the reference airfoil. In addition, the flow reattachment occurs earlier in this DMLE case.

With $A_{D}$ = 0.0075, the lift coefficient of the DMLE airfoil increased by 11.46% as compared to the reference airfoil, and the flow was also reattached earlier for the DMLE airfoil as compared to that of the reference airfoil. This DMLE airfoil also results in reduced drag coefficients as compared to the reference airfoil during both the upstroke and the downstroke cycles.

Similarly, Figure 12b shows the drag coefficients variations with the angle of attack of three DMLE airfoil cases ($A_{D}$ = 0.01, 0.005 and 0.0075) in comparison to those of the reference airfoil. It is seen that the $C_{D,max}$ value is higher for the DMLE airfoil than the $C_{D,max}$ of the reference airfoil. In the downstroke, the $C_{D}$ values are lower than those of the reference airfoil. This fact indicates that by controlling the DMLE airfoil deflection and the morphing starting time, the maximum aerodynamic coefficients can be improved accordingly. The LEVs are formed much earlier in the reference airfoil than in the DMLE airfoil, thus suggesting that the stall angle of attack delay helps to increase the airfoil lift coefficient, even in the downstroke cycle.

Therefore, it can be concluded that among the three droop amplitudes ($A_{D}),$ $A_{D}$ = 0.005 provides the best performance considering the overall aerodynamic efficiency. It is worth noting that the variation of the $M_{ST}$ significantly affects the aerodynamic coefficient values. According to the definition of droop deformation, the droop rate varies with the time steps and therefore, the leading-edge shape also changes accordingly. This conclusion also reveals that the $M_{ST}$ should be studied along with $A_{D}$ to further investigate and improve the overall DMLE performance gains.

**Figure 13:**
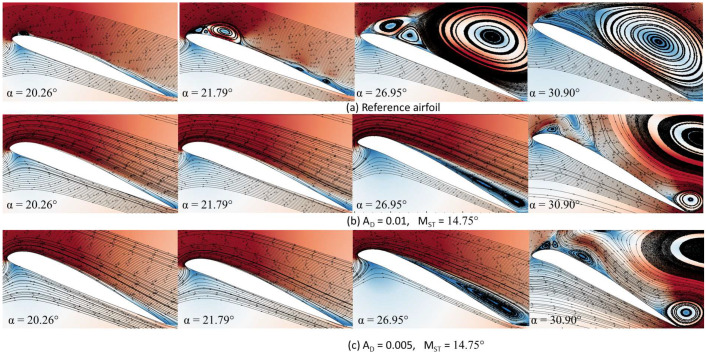
Velocity contours with flow streamlines for the (**a**) reference airfoil, (**b**) DMLE airfoil with $A_{D}$ = 0.01 and (**c**) DMLE airfoil with $A_{D}$ = 0.005.

The reference airfoil’s flow streamlines and velocity contours, and those of airfoils with $A_{D}$ values of 0.01 and 0.005, are all shown in Figure 13a–c, respectively. Figure 13a shows the flow development for the reference airfoil at various upstroke angles of attack. At 20.26°, the tiny LEV of the reference airfoil is seen. The vortex size increases as the angle of attack increases to 21.79°. Along with the primary LEV, secondary, and tertiary LEVs are also formed. A Dynamic Stall Vortex (DSV) occurs as the angle of attack increases between 26.95° and 30.90°; thereby the flow increases through the boundary layer as secondary and tertiary vortices occur.

In the case of DMLE airfoils, as seen in Figure 13b, the flow separation phenomenon is not seen at low angles of attack, such as 20.26° and 21.79°, as the flow remains attached to the airfoil. At an angle of attack of 26.95°, the leading-edge suction increases, the LEV moves towards the trailing edge, and the TEV has formed accordingly. Then, the flow fully separates at an angle of attack of 30.90°. Figure 13c shows the flow phenomena for the DMLE airfoil with $A_{D}=$0.005 at different angles of attack. It shows similar behaviour as observed in the case with $A_{D}$ = 0.01.

Therefore, it can be concluded that the DMLE airfoil cases with different deflection amplitudes improve aerodynamic efficiency ($C_{L,max},\;C_{D,max})$ and delay the dynamic stall angle. The flow remained largely attached to the DMLE airfoils and the separation phenomena associated with a dynamic stall were reduced.

### 3.2. Results for Dynamically Morphing Leading Edge (DMLE) of a Fixed Airfoil

**Figure 14:**
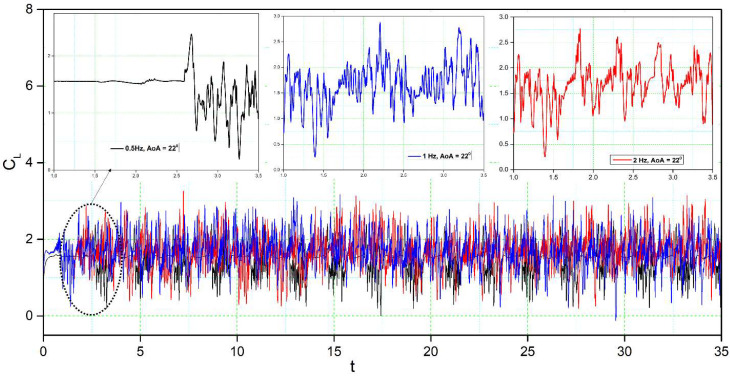
Lift coefficient transient responses for DMLE airfoil at 0.5 Hz, 1 Hz, and 2 Hz.

**Figure 15:**
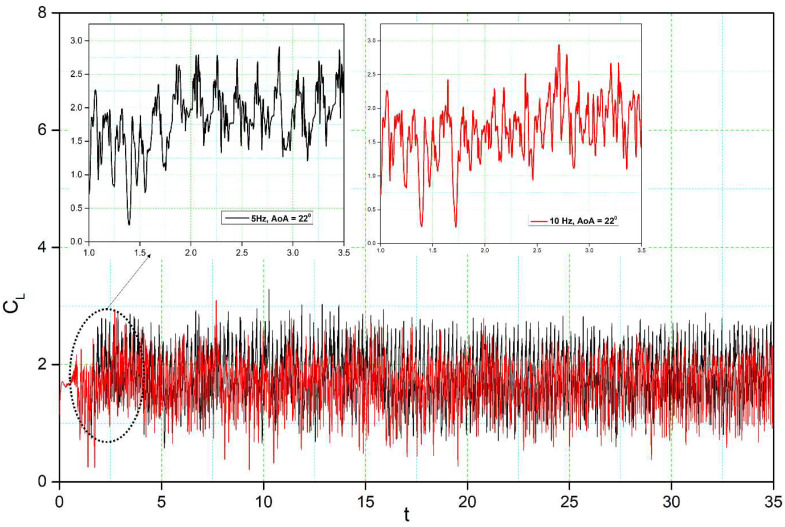
Lift coefficient transient responses for DMLE airfoil at 5 Hz and 10 Hz.

**Figure 16:**
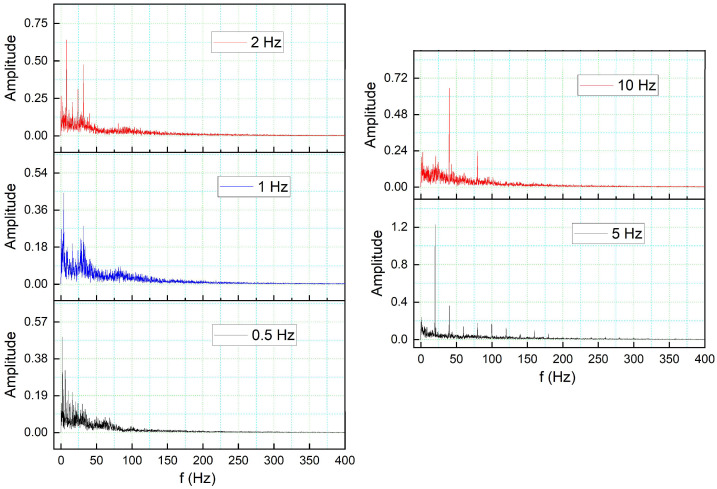
Lift coefficient transient responses for DMLE airfoil at 5 Hz and 10 Hz.

**Figure 17:**
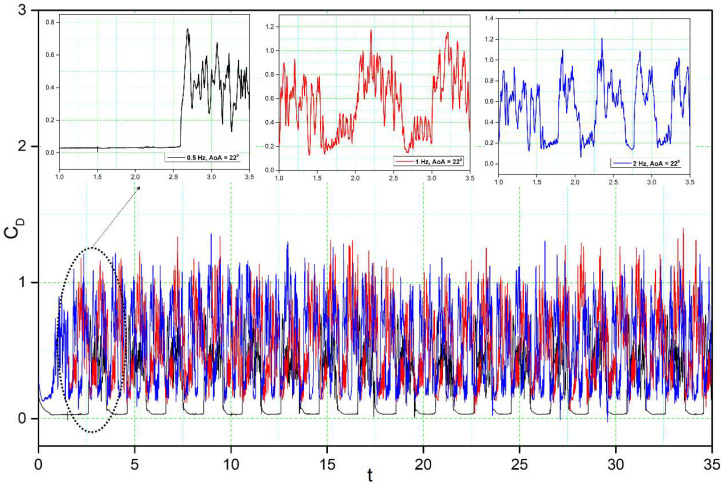
Drag coefficient transient responses for DMLE airfoil at 0.5 Hz, 1 Hz, and 2 Hz.

Figure 14, Figure 15, Figure 16 and Figure 17 show the transient lift and drag coefficient for the DMLE airfoils at five different frequencies at an angle of attack of 22° degrees. This angle of attack was chosen because the flow is fully separated, and major vortices are formed at 22°. For all these simulation cases, the DMLE begins drooping at t = 1.5 s. The transient lift coefficient peaks increase as the leading edge keeps morphing dynamically. At low frequencies, such as 0.5 Hz, 1 Hz, and 2 Hz, the lift slope decreases when the DMLE airfoil begins its morphing until it reaches its maximum deflection. When the DMLE airfoil returns to its reference shape, the lift slope starts to increase again. As the DNLE deflects upwards, the flow separation region increases. Lift coefficient peaks for 1 Hz and 2 Hz frequencies are mostly higher than one and their peak values are between 2.5 and 2.8. For 0.5 Hz, the lift coefficient values are below 1. Similarly, more lift coefficient peaks above the value of 2 are found at 2 Hz than at 1 Hz. As the frequencies increase to 5 and 10 Hz, the DMLE deflection results in a higher number of lift coefficients peaks above 1.5. The higher frequencies conduct more transient flow than the lower frequencies, and therefore, to large flow vortices on the airfoil.

In this study, the upward and downward deflection motions of the DMLE airfoil have shown that the downward deflection of the leading edge increased the stall angle of attack and the nose-down pitching moment. Furthermore, the larger the downward deflection angle, the better the lift-to-drag ratio of the DMLE airfoil will be at a small angle of attack. For the upward deflection, these results were reversed.

The formation of unstable flow over an airfoil is greatly influenced by the development and shedding of vortices, which also have an impact on its aerodynamic characteristics. Analysis of the shedding vortices flow field properties variation in the time-domain, however, is challenging. The discrete Fast Fourier Transform (FFT) algorithm is used to convert the aerodynamic coefficient from the time domain to the frequency domain. Based on the transient lift coefficient for the DMLE airfoil, the amplitude spectrum was estimated using the FFT algorithm; its frequency peak was caused by vortex shedding on the airfoil. Figure 16 shows that the frequency spectrum for a high angle of attack of 18° exhibits a denser and greater-amplitude frequency spectrum for different deflection frequencies. It is well known that the frequency decreases with increasing angle of attack, thus indicating that the shedding period of vortices is increased.

For the DMLE airfoil, the oscillation curve is not periodic, and the lift coefficient curves are disordered, as seen in Figure 14 and Figure 15. The lift coefficient spectrum contains multiple deflection frequencies with different amplitudes, which demonstrates that the angle of attack shedding vortex has an aperiodic structure and that there are numerous vortices with various frequencies and comparable strengths. Similarly, the intensity of vortices uses too much flow field energy in the disturbance that does not contribute too much to the lift coefficient, thus preventing it from increasing further.

**Figure 18:**
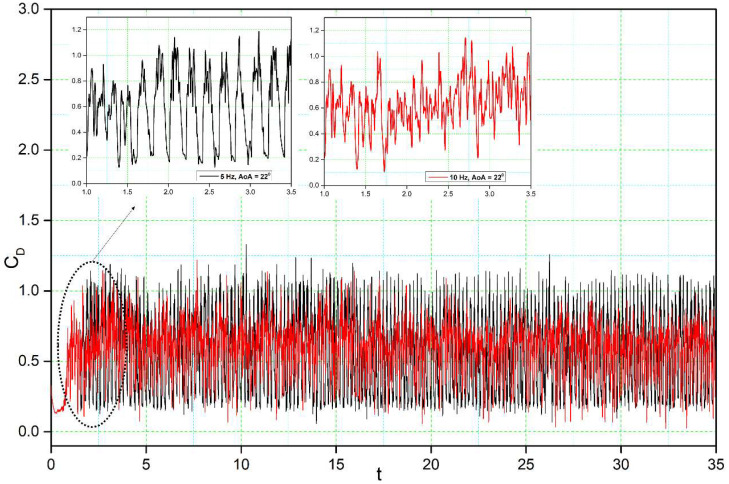
Drag coefficient transient responses for DMLE airfoil at 5 Hz and 10 Hz.

The transient drag response in Figure 17 and Figure 18 shows nearly the same trend as the transient lift coefficient (Figure 14 and Figure 15). Initially, an overshoot is observed in the drag coefficient as the leading edge starts to morph at t = 1.5 s, and the amplitude of the drag is also proportional to the deflection frequency: high deflection frequencies cause large changes in the drag values. The reason for these large overshoots may be due to the sudden pressure changes over the leading edge of the airfoil. The downward deflection of the DMLE results in better aerodynamic efficiency.

**Figure 19:**
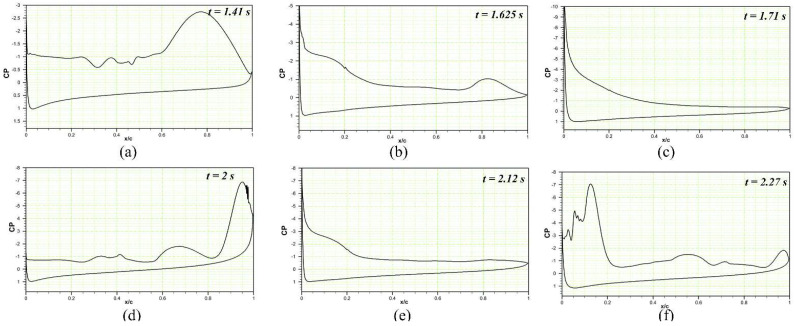
Pressure coefficient variations along the chord comparison of the reference airfoil with the DMLE airfoil at the angle of attack of 22° at the deflection frequency of 2 Hz for (**a**) time step of 1.41 s, (**b**) time step of 1.625 s, (**c**) time step of 1.71 s, (**d**) time step of 2 s, (**e**) 2.12 s and (**f**) time step of 2.27 s. These figures reveal the pressure coefficients at different morphing leading edge deflections and how the flow stability increases by minimizing the flow separation over an airfoil.

**Figure 20:**
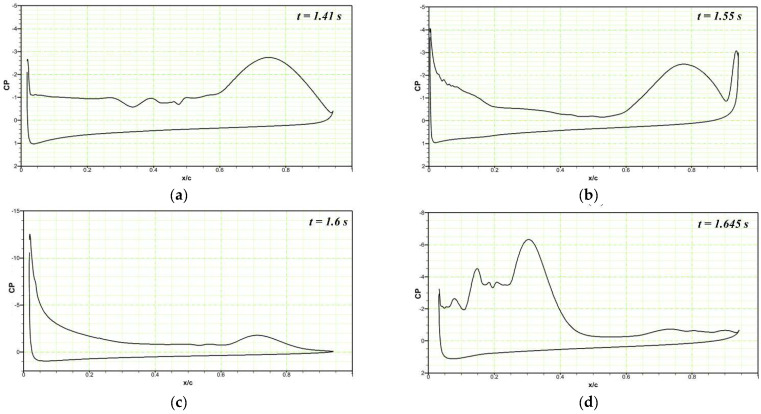
Pressure coefficient variations along the chord comparison of the reference airfoil with the DMLE airfoil at the angle of attack of 22° at the deflection frequency of 5 Hz for (**a**) time step of 1.41 s, (**b**) time step of 1.55 s, (**c**) time step of 1.6 s, (**d**) time step of 1.645 s. These figures reveal the pressure coefficients at different morphing leading edge deflections and also the deflection is faster at higher frequencies.

**Figure 21:**
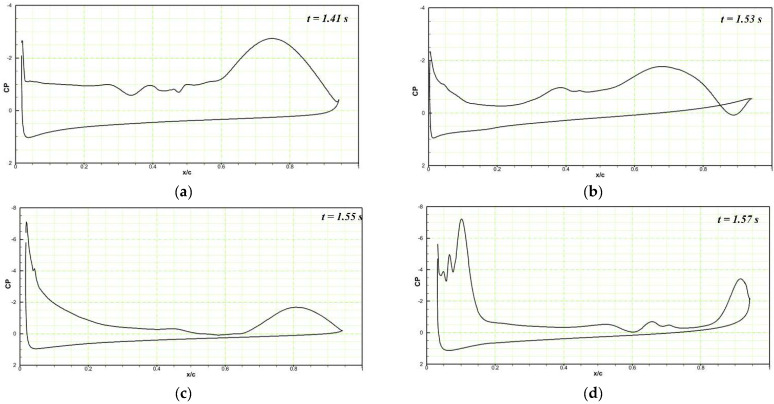
Pressure coefficients variations along the chord comparison of the reference airfoil with the DMLE airfoil at the angle of attack of 22 at the deflection frequency of 10 Hz for (**a**) time step of 1.41 s, (**b**) time step of 1.53 s, (**c**) time step of 1.55 s, (**d**) time step of 1.57 s. These figures reveal the pressure coefficients at different morphing leading edge deflections and also the deflection is faster at higher frequencies.

The pressure coefficients of the DMLE airfoil at different frequencies are shown in Figure 19, Figure 20 and Figure 21. Figure 19 shows that at an angle of attack of 22° for the deflection frequency of 2 Hz and at the time, t = 1.41 s, the airfoil is already in a pre-stall condition, and has large LEVs over the airfoil; however, the flow remains attached to the DMLE airfoil. The flow is characterized by the presence of a large vortex near the trailing edge of an airfoil. The leading-edge morphing starts at t = 1.5 s and continues to deflect downwards until it reaches the maximum deflection at t = 1.71 s. It can be seen that at t = 1.625 s and t = 1.71 s, the flow remains largely attached to the airfoil, and does not have any significant LEVs over the surface. The leading edge starts to move upwards back to its original position while the pressure drops significantly, and a large trailing edge vortex separation can be seen at t = 2 s. The flow becomes stable again at t = 2.12 s and separates at t = 2.27 s. This process reveals that the downward deflection of the leading edge increases the flow stability and increases the stall angle of attack by delaying the formation of a Dynamic Stall Vortex (DSV).

At the deflection frequency of 5 Hz, the DMLE droops at a faster rate compared to its behavior at low frequencies such as 0.01 Hz and 0.005 Hz. Figure 20a shows the chordwise pressure distribution at t = 1.41 s; LEVs can be observed over the airfoil. At t = 1.55 s, the leading-edge droops to the maximum value, and a large TEV can be seen over the airfoil (Figure 20b). When the leading edge starts to return to its original position, the flow stabilizes and reattaches with the airfoil, as seen at t = 1.6 s (Figure 20c). The flow takes more time to reattach to the airfoil at high frequencies as compared to low-frequency deflections. The leading edge continues to deflect upwards, and the large LEV and flow separation can be seen at t = 1.645 s (Figure 20d). Similar type outcomes are obtained for the deflection frequency of 10 Hz, as shown in Figure 21a–d.

**Figure 22:**
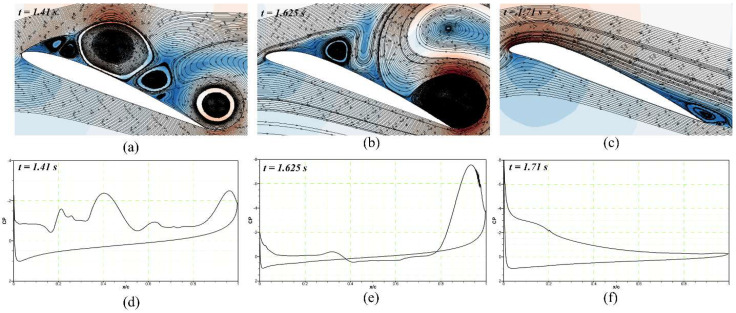
The downward deflection of the DMLE airfoil shows the (**a**) velocity streamline contours at time step of 1.41 s, (**b**) velocity streamline contours at time step of 1.625 s, (**c**) velocity streamline contours at time step of 1.71 s, (**d**) pressure coefficient at time step of 1.41 s, (**e**) pressure coefficient at time step 1.625 s and (**f**) pressure coefficient at time step of 1.71 s. These figures reveal the velocity stream lines with their respective pressure coefficients at different morphing leading edge deflections and how the flow stability increases by minimizing the flow separation over an airfoil.

To depict the vortices for the DMLE at the deflection frequency of 1 Hz and an angle of attack of 22° at different time steps, Figure 22a shows that before the starting of morphing at t = 1.41 s, a series of vortices are observed over the airfoil, which may be expressed as their respective pressure distribution in Figure 22d. As the DMLE morphing starts at t = 1.5 s, the DMLE starts to droop, therefore the leading edge of the airfoil changes. Finally, at t = 1.625 s, the pressure distribution graph reveals that a reverse flow region arises and moves towards the trailing edge, as shown in Figure 22b,e. Keeping track of the vortices’ formation, growth, and destruction and analyzing their magnitude is one of the important methods to evaluate the dynamic stall process. However, even with the best visual aids, flow circulation values can occasionally vary noticeably, independently of vortices sizes. Therefore, further data analysis, such as looking at pressure distributions at the wall and in the area around it, must be conducted.

**Figure 23:**
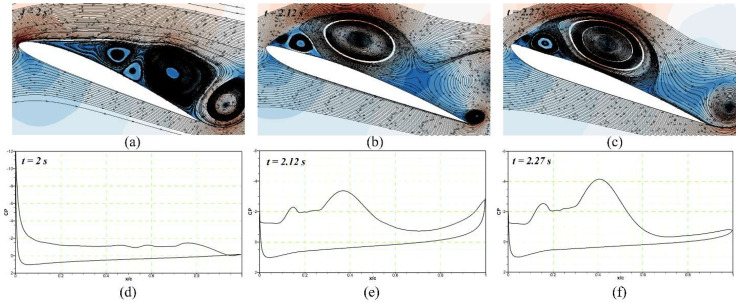
The upward deflection of the DMLE airfoil shows the (**a**) velocity streamline contours at time step of 2 s, (**b**) velocity streamline contours at time step of 2.12 s, (**c**) velocity streamline con-tours at time step of 2.27 s, (**d**) pressure coefficient at time step of 2 s, (**e**) pressure coefficient at time step 2.12 s and (**f**) pressure coefficient at time step of 2.27 s. These figures reveal the velocity stream lines with their respective pressure coefficients at different morphing leading edge de-flections and how the flow stability increases by minimizing the flow separation over an airfoil.

The droop nose reaches its maximum deflection at t = 1.71 s, as shown in Figure 22c, and it remains attached to the airfoil (Figure 22f). Then, the airfoil returns to its original shape by deflecting the DMLE upwards. At t = 2 s, the vortices and a predominant flow-recirculating zone appear in the vicinity of the trailing edge region (Figure 23a,c). As the airfoil deflects upwards, stronger LEVs are formed, as seen in Figure 23b,e. By observing the Spatio-temporal evolution of both LEVs, at t = 2.27 s (Figure 23c,f), it is possible to understand how the fast-rolling up the mechanism of the trailing edge shear layer and the LEV keep growing due to the kinetic energy; this energy is supplied by the LEV gradient as a small counter-rotating vortex starts to form between the leading-edge and the LEV. In this full cycle process, it is evident that the flow remains attached to the airfoil when the DMLE is deflected downwards, and therefore, the DSV control can also be conducted by the droop nose morphing process.

## 4. Conclusions

This paper investigated the effect of the DMLE on the flow structure and behavior of vortices around a pitching UAS-S45 airfoil during the dynamic stall. The study focused on: (1) developing a framework to dynamically morph the leading edge of the S45 airfoil through a camber line deformation, and then (2) analyzing the aerodynamic performance in terms of controlling the dynamic stall phenomenon. The framework was developed using an unsteady parametrization method from the UAS-S45 airfoil parametric equations, specifically adapted to obtain the morphing motion of the leading edge over time. This scheme was then integrated within the ANSYS Fluent solver by developing a User-Defined-Function (UDF) code to dynamically deflect the airfoil boundaries and control the dynamic mesh used for its deformation and adaption. The dynamic and sliding mesh techniques were used to simulate the unsteady flow across the sinusoidally pitching UAS-S45 airfoil. The $\gamma -Re_{\theta }$ turbulence model is used due to its ability to capture the flow structures of dynamic airfoils.

Two main studies were considered: (i) firstly, an investigation of the Dynamically Morphing Leading-Edge (DMLE) of an oscillating airfoil. In this study, the pitching-oscillation motion of an airfoil was defined, and its parameters, such as its droop nose amplitude and morphing starting time, were evaluated. The effects of both parameter variations of DMLE on the aerodynamic performance were studied, and three different amplitudes were considered; (ii) secondly, the DMLE of an airfoil at the stall angles of attack was investigated. The DMLE performance was analyzed to provide further insights into the dynamic lift and drag force variations at pre-defined deflection frequencies of 1 Hz, 2 Hz, 5 Hz, and 10 Hz. Some significant conclusions are outlined below:

- The unsteady aerodynamic parametrization method coupled with Laplace Diffusion dynamic mesh techniques gave good results. The mesh quality metrics were very well respected during the entire deformation process; hence, an accurate simulation process was confirmed by the validation of the results and mesh deformation schemes.
- For the DMLE of an oscillating airfoil, when $A_{D}$ = 0.01 and $M_{ST}$ = 14.75°, the lift coefficient increased by 20.15%, while a 16.58% delay in the dynamic stall angle was obtained compared to the reference airfoil. Similarly, the lift coefficients obtained for the two other cases, when $A_{D}$ = 0.05 and $A_{D}$ = 0.0075, increased by 10.67% and 11.46%, respectively, compared to the reference airfoil.
- The presence of a LEV was depicted in the case of the reference airfoil at the angle of attack of 21.97°, also seen as a “bump” in the surface pressure distribution. By the time the angle of attack reaches 26.95°, the LEV increased and spread over the large part of the airfoil. However, in the case of the DMLE airfoils with $A_{D}$ = 0.01, 0.005, and 0075, no strong leading-edge vortex was observed for the same angles of attack of the reference airfoil.
- The numerical results have shown that the new radius of curvature of the DMLE airfoil can minimize the streamwise adverse pressure gradient, and further prevent significant flow separation by delaying the Dynamic Stall Vortex (DSV) occurrence. Furthermore, it was shown that the DMLE airfoil delayed the stall angle of attack with respect to the reference airfoil by 16.58%.
- In the case of the DMLE of an airfoil at a given angle of attack, the lift slope decreases as the leading-edge morphing begins until it reaches the maximum deflection at low deflection frequencies. When the DMLE returns to its original position, the lift slope increases again. The leading edge deflects upwards, resulting in increased flow separation and high lift slopes. The DMLE repeats the cycle, and the same trend is followed by the lift and drag coefficients of the DMLE airfoil.
- The DMLE deflects rapidly at higher frequencies, such as 5 Hz and 10 Hz, resulting in increased lift coefficients. The higher frequencies lead to more transient flow; therefore, the flow remains separated from the airfoil. In this study, the upward and downward deflection motions of DMLE airfoils have shown that the downward deflection of the DMLE increases the stall angle of attack and the nose-down pitching moment. Furthermore, the larger the downward deflection angle, the higher the lift-to-drag of the morphing wing.

Regarding future works, the LARCASE’s Price-Padoussis subsonic wind tunnel will be used for future wind tunnel studies of the DMLE airfoils. The findings are expected to clarify the flow physics, and hence validate the findings of the unsteady flow behaviour of the DMLE airfoil.

## Figures and Tables

**Table 1:** Grid properties of the three grid sizes for the grid-sensitivity analysis.

Grid Size	Number of Cells	Min Length	Max Length	Bias Factor
1	62 626	0.001	0.06	1.12
2	103 212	0.001	0.035	1.08
3	206 038	0.001	0.02	1.05

## Data Availability

The data presented in this study are available on request from the corresponding author.

## Nomenclature

α	Angle of attack
$\alpha_{m}$	Mean incidence angle
$\alpha_{a}$	Amplitude incidence angle
$A_{D}$	Droop nose deflection
$C_{L}$	Lift coefficient
$C_{L,max}$	Maximum lift coefficient
$C_{D}$	Drag coefficient
$C_{D,max}$	Maximum drag coefficient
c	Chord
$C_{P}$	Pressure coefficient
k	Reduced frequency
M	Maximum value of the percentage chord line
$M_{ST}$	Morphing starting time
P	Chordwise position of the maximum camber
t	Time
$U_{\infty }$	Freestream velocity
$W_{le}$	Value of the maximum deflection of the leading edge
$y_{f}$	Final y-coordinate of the new morphing airfoil camber line
*LEV*	Leading edge vortex
*TEV*	Trailing edge vortex
*DSV*	Dynamic stall vortex
*DMLE*	Dynamically morphing leading edge
*UDF*	User-defined function
